# Supplementary material for: Bicuspid Aortic Valve: Old and Novel Gene Contribution to Disease Onset and Complications
Source: Diagnostics (Basel). 2025 Dec 28;16(1):104. doi: 10.3390/diagnostics16010104 (PMC12785301; doi:10.3390/diagnostics16010104)
Supplement: Supplementary file 1 [file diagnostics-16-00104-s001.zip › Supplementary files/Table 1 Supplementary Diagnostics.pdf]

Supplementary Table 1. Custom targeted 97 genes NGS panel details

Gene	Name	Position	OMIM	Reference
COL11A1	COLLAGEN, TYPE XI, ALPHA-1	1p21.1	* 120280	NM_001854
TGFBR3	TRANSFORMING GROWTH FACTOR-BETA RECEPTOR, TYPE III;	1p22.1	* 600742	NM_003243
MFAP2	MICROFIBRILLAR-ASSOCIATED PROTEIN 2	1p36.13	* 156790	NM_002403
MTHFR	5,10-METHYLENETETRAHYDROFOLATE REDUCTASE	1p36.22	* 607093	NM_005957
PLOD1	PROCOLLAGEN-LYSINE, 2-OXOGLUTARATE 5-DIOXYGENASE	1p36.22	* 153454	NM_000302
SKI	V-SKI AVIAN SARCOMA VIRAL ONCOGENE HOMOLOG	1p36.33-p36.32	* 164780	NM_003036
B3GALT6	BETA-1,3-GALACTOSYLTRANSFERASE 6	1p36.33	* 615291	NM_080605
ADAMTSL4	ADAMTS-LIKE 4	1q21.2	* 610113	NM_019032
PTGS2	PROSTAGLANDIN-ENDOPEROXIDE SYNTHASE 2	1q31.1	* 600262	NM_000963
TGFB2	TRANSFORMING GROWTH FACTOR, BETA-2	1q41	* 190220	NM_001135599
CAPN2	CALPAIN 2	1q41	* 114230	NM_001748
AGT	ANGIOTENSINOGEN	1q42.2	* 106150	NM_001384479
MTR	5-METHYLTETRAHYDROFOLATE-HOMOCYSTEINE S-METHYLTRANSFERASE	1q43	* 156570	NM_000254
LTBP1	LATENT TRANSFORMING GROWTH FACTOR-BETA-BINDING PROTEIN 1	2p22.3	* 150390	NM_206943
EMILIN1	ELASTIN MICROFIBRIL INTERFACER 1	2p23.3	* 130660	NM_007046
MMADHC	MMADHC GENE	2q23.2	* 611935	NM_015702
ACVR1	ACTIVIN A RECEPTOR, TYPE I	2q24.1	* 102576	NM_001111067
COL3A1	COLLAGEN, TYPE III, ALPHA-1	2q32.2	* 120180	NM_000090
COL5A2	COLLAGEN, TYPE V, ALPHA-2	2q32.2	* 120190	NM_000393
FN1	FIBRONECTIN 1	2q35	* 135600	NM_212482
COL6A3	COLLAGEN, TYPE VI, ALPHA-3	2q37.3	* 120250	NM_004369
TGFBR2	TRANSFORMING GROWTH FACTOR-BETA RECEPTOR, TYPE II	3p24.1	* 190182	NM_001024847
VHL	VHL GENE	3p25.3	* 608537	NM_000551
ZPLD1	ZONA PELLUCIDA-LIKE DOMAIN-CONTAINING PROTEIN 1	3q12.3	* 615915	NM_175056
MYLK	MYOSIN LIGHT CHAIN KINASE	3q21.1	* 600922	NM_053025
AGTR1	ANGIOTENSIN RECEPTOR 1	3q24	* 106165	NM_004835
PDCD10	PROGRAMMED CELL DEATH 10	3q26.1	* 609118	NM_145860
MTRR	METHIONINE SYNTHASE REDUCTASE	5p15.31	* 602568	NM_024010
AGGF1	ANGIOGENIC FACTOR WITH G-PATCH AND FHA DOMAINS 1	5q13.3	* 608464	NM_018046
FBN2	FIBRILLIN 2	5q23.3	* 612570	NM_001999
NKX2-5	NK2 HOMEOBOX 5	5q35.1	* 600584	NM_004387
B4GALT7	BETA-1,4-GALACTOSYLTRANSFERASE 7	5q35.3	* 604327	NM_007255
ADAMTS2	A DISINTEGRIN-LIKE AND METALLOPROTEINASE WITH THROMBOSPONDIN TYPE 1 MOTIF, 2	5q35.3	* 604539	NM_014244
COL11A2	COLLAGEN, TYPE XI, ALPHA-2	6p21.32	* 120290	NM_080680
TNXB	TENASCIN XB	6p21.33-p21.32	* 600985	NM_019105
COL9A1	COLLAGEN, TYPE IX, ALPHA-1	6q13	* 120210	NM_001851
DSE	DERMATAN SULFATE EPIMERASE	6q22.1	* 605942	NM_001080976
CCM2	CCM2 GENE	7p13	* 607929	NM_001029835
FKBP14	FK506-BINDING PROTEIN 14	7p14.3	* 614505	NM_017946
HOXA1	HOMEOBOX A1	7p15.2	* 142955	NM_005522
ELN	ELASTIN	7q11.23	* 130160	NM_000501
KRIT1	KREV INTERACTION TRAPPED	7q21.2	* 604214	NM_194456
COL1A2	COLLAGEN, TYPE I, ALPHA-2	7q21.3	* 120160	NM_000089
NOS3	NITRIC OXIDE SYNTHASE 3	7q36.1	+ 163729	NM_000603
GNAQ	GUANINE NUCLEOTIDE-BINDING PROTEIN, Q POLYPEPTIDE	9q21.2	* 600998	NM_002072
TGFBR1	TRANSFORMING GROWTH FACTOR-BETA RECEPTOR, TYPE I	9q22.33	* 190181	NM_004612
PTGS1	PROSTAGLANDIN-ENDOPEROXIDE SYNTHASE 1	9q33.2	* 176805	NM_000962
ENG	ENDOGLIN	9q34.11	* 131195	NM_001114753
COL5A1	COLLAGEN, TYPE V, ALPHA-1	9q34.3	* 120215	NM_001278074
NOTCH1	NOTCH, DROSOPHILA, HOMOLOG OF, 1	9q34.3	* 190198	NM_017617
RET	REARRANGED DURING TRANSFECTION PROTOONCOGENE	10q11.21	* 164761	NM_020975
ACTA2	ACTIN, ALPHA-2, SMOOTH MUSCLE, AORTA	10q23.31	* 102620	NM_001613
FGF8	FIBROBLAST GROWTH FACTOR 8	10q24.32	* 600483	NM_033163
B3GAT3	BETA-1,3-GLUCURONYLTRANSFERASE 3	11q12.3	* 606374	NM_012200
LTBP3	LATENT TRANSFORMING GROWTH FACTOR-BETA-BINDING PROTEIN 3	11q13.1	* 602090	NM_001130144
EFEMP2/fbln4	EGF-CONTAINING FIBULIN-LIKE EXTRACELLULAR MATRIX PROTEIN 2	11q13.1	* 604633	NM_016938
LRP5	LOW DENSITY LIPOPROTEIN RECEPTOR-RELATED PROTEIN 5	11q13.2	* 603506	NM_002335
CCND1	CYCLIN D1	11q13.3	* 168461	NM_053056
LRP6	LOW DENSITY LIPOPROTEIN RECEPTOR-RELATED PROTEIN 6	12p13.2	* 603507	NM_002336
COL2A1	COLLAGEN, TYPE II, ALPHA-1	12q13.11	* 120140	NM_001844
LRP1	LOW DENSITY LIPOPROTEIN RECEPTOR-RELATED PROTEIN 1	12q13.3	* 107770	NM_002332
DCN	DECORIN	12q21.33	* 125255	NM_133503

LTBP2	LATENT TRANSFORMING GROWTH FACTOR-BETA-BINDING PROTEIN 2	14q24.3	* 602091	NM_000428
TGFB3	TRANSFORMING GROWTH FACTOR, BETA-3	14q24.3	* 190230	NM_003239
FBLN5	FIBULIN 5	14q32.12	* 604580	NM_006329
CHST14	CARBOHYDRATE SULFOTRANSFERASE 14	15q15.1	* 608429	NM_130468
FBN1	FIBRILLIN 1	15q21.1	* 134797	NM_000138
SMAD3	MOTHERS AGAINST DECAPENTAPLEGIC, DROSOPHILA, HOMOLOG OF, 3	15q22.33	* 603109	NM_005902
ADAMTS17	A DISINTEGRIN-LIKE AND METALLOPROTEINASE WITH THROMBOSPONDIN TYPE 1 MOTIF	15q26.3	* 607511	NM_139057
MAPK3	MITOGEN-ACTIVATED PROTEIN KINASE 3	16p11.2	* 601795	NM_002746
MYH11	MYOSIN, HEAVY CHAIN 11, SMOOTH MUSCLE	16p13.11	* 160745	NM_001040114
ABCC6	ATP-BINDING CASSETTE, SUBFAMILY C, MEMBER 6	16p13.11	* 603234	NM_001171
PDIA2	PROTEIN DISULFIDE ISOMERASE, FAMILY A, MEMBER 2	16p13.3	* 608012	NM_006849
AXIN1	AXIS INHIBITOR 1	16p13.3	* 603816	NM_003502
MMP2	MATRIX METALLOPROTEINASE 2	16q12.2	* 120360	NM_004530
CRYBA1	CRYSTALLIN, BETA-A1	17q11.2	* 123610	NM_005208
COL1A1	COLLAGEN, TYPE I, ALPHA-1	17q21.33	* 120150	NM_000088
ACE	ANGIOTENSIN I-CONVERTING ENZYME	17q23.3	+ 106180	NM_000789
KCNJ2	POTASSIUM CHANNEL, INWARDLY RECTIFYING, SUBFAMILY J, MEMBER 2	17q24.3	* 600681	NM_000891
EMILIN2	ELASTIN MICROFIBRIL INTERFACER 2	18p11.32	* 608928	NM_032048
SMAD2	MOTHERS AGAINST DECAPENTAPLEGIC, DROSOPHILA, HOMOLOG OF, 2	18q21.1	* 601366	NM_001003652
SMAD4	MOTHERS AGAINST DECAPENTAPLEGIC, DROSOPHILA, HOMOLOG OF, 4	18q21.2	* 600993	NM_005359
ADAMTS10	A DISINTEGRIN-LIKE AND METALLOPROTEINASE WITH THROMBOSPONDIN TYPE 1 MOTIF, 10	19p13.2	* 608990	NM_030957
LTBP4	LATENT TRANSFORMING GROWTH FACTOR-BETA-BINDING PROTEIN 4	19q13.2	* 604710	NM_001042544
TGFB1	TRANSFORMING GROWTH FACTOR, BETA-1	19q13.2	* 190180	NM_000660
JAG1	JAGGED 1	20p12.2	* 601920	NM_000214
EMILIN3	ELASTIN MICROFIBRIL INTERFACER 3	20q12	* 608929	NM_052846
MMP9	MATRIX METALLOPROTEINASE 9	20q13.12	* 120361	NM_004994
SLC2A10	SOLUTE CARRIER FAMILY 2 (FACILITATED GLUCOSE TRANSPORTER), MEMBER 10	20q13.12	* 606145	NM_030777
GATA5	GATA-BINDING PROTEIN 5	20q13.33	* 611496	NM_080473
CBS	CYSTATHIONINE BETA-SYNTHASE	21q22.3	* 613381	NM_001178008
COL6A1	COLLAGEN, TYPE VI, ALPHA-1	21q22.3	* 120220	NM_001848
COL6A2	COLLAGEN, TYPE VI, ALPHA-2	21q22.3	* 120240	NM_001849
UFD1L	UBIQUITIN FUSION DEGRADATION 1-LIKE	22q11.21	* 601754	NM_005659
MAPK1	MITOGEN-ACTIVATED PROTEIN KINASE 1	22q11.22	* 176948	NM_002745
AGTR2	ANGIOTENSIN II RECEPTOR, TYPE 2	Xq23	* 300034	NM_000686
FLNA	FILAMIN A	Xq28	* 300017	NM_001110556
